# A Comprehensive Review for Removal of Non-Steroidal Anti-Inflammatory Drugs Attained from Wastewater Observations Using Carbon-Based Anodic Oxidation Process

**DOI:** 10.3390/toxics10100598

**Published:** 2022-10-10

**Authors:** Zainab Haider Mussa, Fouad Fadhil Al-Qaim, Ali H. Jawad, Miklas Scholz, Zaher Mundher Yaseen

**Affiliations:** 1 College of Pharmacy, University of Al-Ameed, Kerbala 56001, Iraq; 2 College of Medicine, University of Warith Al-Anbiyaa, Karbala 56001, Iraq; 3 Department of Chemistry, College of Science for Women, University of Babylon, Hillah 51001, Iraq; 4 Faculty of Applied Sciences, Universiti Teknologi MARA, Shah Alam 40450, Selangor, Malaysia; 5 Directorate of Engineering the Future, School of Science, Engineering and Environment, The University of Salford, Newton Building, Salford M5 4WT, Greater Manchester, UK; 6 Department of Civil Engineering Science, School of Civil Engineering and the Built Environment, University of Johannesburg, Kingsway Campus, Johannesburg 2092, South Africa; 7 Department of Town Planning, Engineering Networks and Systems, South Ural State University (National Research University), 76, Lenin Prospekt, 454080 Chelyabinsk, Russia; 8 Civil and Environmental Engineering Department, King Fahd University of Petroleum & Minerals, Dhahran 31261, Saudi Arabia

**Keywords:** NSAIDs presence in wastewater and water, wastewater treatment technologies, electrochemical process, carbon-based anode, byproducts

## Abstract

Non-steroidal anti-inflammatory drugs (NSAIDs) (concentration <µg/L) are globally acknowledged as hazardous emerging pollutants that pass via various routes in the environment and ultimately enter aquatic food chains. In this context, the article reviews the occurrence, transport, fate, and electrochemical removal of some selected NSAIDs (diclofenac (DIC), ketoprofen (KTP), ibuprofen (IBU), and naproxen (NPX)) using carbon-based anodes in the aquatic environment. However, no specific protocol has been developed to date, and various approaches have been adopted for the sampling and elimination processes of NSAIDs from wastewater samples. The mean concentration of selected NSAIDs from different countries varies considerably, ranging between 3992–27,061 µg/L (influent wastewater) and 1208–7943 µg/L (effluent wastewater). An assessment of NSAIDs removal efficiency across different treatment stages in various wastewater treatment plants (WWTPs) has been performed. Overall, NSAIDs removal efficiency in wastewater treatment plants has been reported to be around 4–89%, 8–100%, 16–100%, and 17–98% for DIC, KTP, NPX, and IBU, respectively. A microbiological reactor (MBR) has been proclaimed to be the most reliable treatment technique for NSAIDs removal (complete removal). Chlorination (81–95%) followed by conventional mechanical biological treatment (CMBT) (94–98%) treatment has been demonstrated to be the most efficient in removing NSAIDs. Further, the present review explains that the electrochemical oxidation process is an alternative process for the treatment of NSAIDs using a carbon-based anode. Different carbon-based carbon anodes have been searched for electrochemical removal of selected NSAIDs. However, boron-doped diamond and graphite have presented reliable applications for the complete removal of NSAIDs from wastewater samples or their aqueous solution.

## 1. Introduction

**Figure 1:**
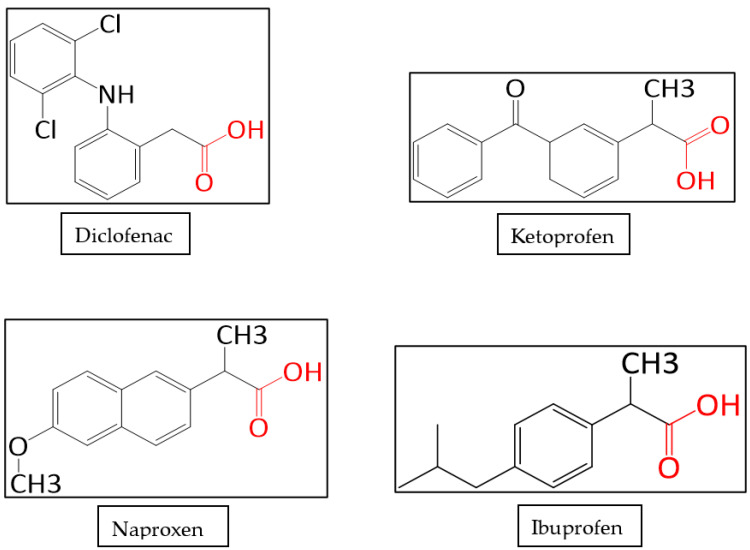
Chemical structure of diclofenac, ketoprofen, naproxen, and ibuprofen.

In 1899, the German Bayer Company was the first company to register the first non-steroidal anti-inflammatory drug, aspirin (acetylsalicylic acid, C_9_H_8_O_4_), followed by many more [1]. Nowadays, more than hundreds of NSAIDs have been synthesized and developed, known as inflammatory reducers and pain-killer drugs. The most commonly known NSAIDs, as reviewed in the present study, are diclofenac, ketoprofen, naproxen, and ibuprofen. They consist of a carboxylic group attached to an aromatic ring, as shown in Figure 1. Non-inflammatory drugs participate in inhibiting the cyclooxygenase enzymes, then reducing inflammation, pain, and fever. Generally, pharmaceuticals consumption is varied from country to country based on the population, common diseases, and so on. However, in Malaysia, about 4.3 prescriptions of non-steroidal inflammatory drugs were consumed in terms of “DDD/inhabitant/year” in 2016 (MOH). In comparison with other countries, ibuprofen and diclofenac have consumption rates reaching up to 37 and 22 tons, respectively, in England, while the rate of consumption was 240, 37, and 22 tons for ibuprofen, naproxen, and diclofenac, respectively, in France [2].

Many NSAID pharmaceuticals are released into aquatic environments continuously due to a significant percentage of unused pharmaceuticals and also due to unsuccessful wastewater treatment, which does not eliminate the amount of NSAIDs released in the aquatic environment. Actually, many sources, such as effluent from sewage treatment plants, industrial companies, domestic wastewater, and effluent from hospitals, are the main source of contamination in surface water [3,4,5,6]. NSAIDs reach the wastewater through the sewer. It is well known that these drugs are partially metabolized in humans, so they are considered highly excreted in the sewer system. Another potential to transfer the pharmaceutical into wastewater systems is the validity of the drug. This means some expired pharmaceuticals ended up directly in the toilet, then reaching the sewer system as well. It was reported that NSAIDs are present in rivers at low concentrations because they were not treated efficiently in WWTPs [7,8].

**Scheme 1:**
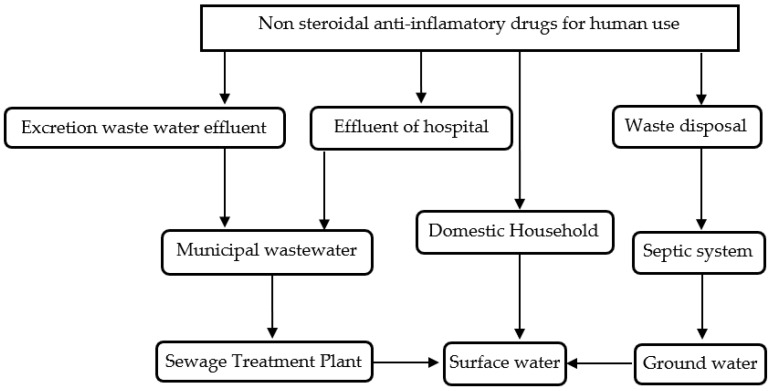
Diagram of sources of NSAIDs pollution.

As we know, NSAIDs may reach directly into wastewater treatment plants via toilets into sewers, then to the surface water, as shown in Scheme 1. However, many NSAIDs could be present in aquatic environment as origin form or metabolized in small percentage. As an example, in Germany, about 60–80% of 16,000 tons of prescribed and non-prescribed pharmaceuticals are disposed of each year from human medical care. However, the main source of transferring these compounds into water is the flushing via toilets [9,10]. Some pharmaceuticals are not treated efficiently, so it escapes into surface water without any treatment. The reason is related to its being highly soluble in water and also not suitable to be degraded using biological or conventional chemical oxidation processes [11,12]. Therefore, NSAIDs have different ways of entering the water sample, and then they have a large impact on it. For example, bank filtration, which is used to receive the groundwater, is considered the main source of NSAIDs entering the drinking water cycle [13,14,15,16]. NSAIDs are detected in a range of nanograms per liter in the effluent of sewage treatment plants and river water [17,18].

It is well known that NSAIDs are released into the surface water as origin compounds because they resist biological degradation, so they keep their chemical structure the same in the human body; however, in this case, it affects aquatic organisms even at low concentrations [19,20,21]. It was reported by the World Health Organization (WHO) that pharmaceuticals could be accumulated and then cause risk to humans and also may pose a potential risk for the organisms living in terrestrial and aquatic environments, so the alarm was begun from continuous releasing these compounds into water [22]. Ibuprofen, naproxen, diclofenac, and ketoprofen as inflammatory drugs have been considered an emerging environmental issue. However, they were present in effluent wastewater and surface water, causing adverse effects on living organisms [23]. In one reported study, it was confirmed that many vultures have been lost or killed due to their exposure to one of the NSAIDs drugs, especially diclofenac residues [24,25].

The co-existence of pharmaceuticals and other chemicals (drug “cocktail”) is one of the reasons for the toxicity in living organisms [26]. It was reported that the combination of diclofenac, ibuprofen, naproxen, and acetylsalicylic acid in water brings more complex toxicity to living organisms [27]. Toxicity issues regarding the presence of pharmaceuticals in water lead to thinking, considering, and developing the improvement in treatment efficiency and also focusing on the assessment of safe drinking water, reclaimed, reused wastewater, and aquatic ecosystems in order to meet the standards of drinking water.

NSAIDs can accumulate in the organs of fish and other living organisms. However, diclofenac and its metabolites were detected in the liver and bile of trout at concentration factors reaching up to 27,000 ng/L as well as in a bioconcentration ranging between 320 and 950 ng/L due to its accumulation ability as a result of chronic exposure [28].

It was reported that ibuprofen, naproxen, and diclofenac have been detected in the bile of wild fish at a concentration of 15 to 34 ng/mL, 6–103 ng/mL, and 6–148 ng/mL, respectively [29]. Consequently, human health could be at risk after consuming fish. It was reported that the metabolites of diclofenac have no toxic effect on lipid peroxidation, reduced glutathione, glutathione-S-transferase, superoxide dismutase, and catalase during the experiments on mice, while the metabolites of ibuprofen have significant toxic effects higher than the parent compound [30,31]. The European Environmental Agency named diclofenac a “contaminant of emerging concern”, so the maximum allowable concentration of diclofenac was limited to 0.1 ng/L in freshwaters and 0.01 μg/L in marine waters. In the United Kingdom, diclofenac brings great attention to developing new technologies to reduce its impact on wastewater samples. However, ibuprofen, naproxen, or ketoprofen have not faced any issues as they exhibited less toxic effects compared with diclofenac because conventional wastewater treatment is not efficient for the elimination of pharmaceuticals or is sometimes poor [4]. However, many countries witnessed pollution of their aquatic environment, so it is considered an aquatic environment issue, and then encouraged to establish research known as “pharmaceutical threaten aquatic environment” [32].

The present review paper exhibited a quick overview and discussion for the remediation of more frequent detection NSAIDs drugs in water bodies namely diclofenac, ketoprofen, naproxen and ibuprofen using electrochemical anodic oxidation processes applying carbon-based anode. The electrochemical oxidation treatment process is still in the first stages compared with other advanced oxidation processes such as ozonation, Fenton, or UV/H_2_O_2_.

The main objectives of this review are to: (i) compare the approaches adopted for NSAIDs occurrence in different wastewater samples, (ii) assess the NSAIDs removal efficacy in different wastewater treatment plants, (iii) apply an electrochemical process using a carbon-based anode on the remediation of the selected NSAIDs.

## 2. The Exhibited Literature Review on NSAIDs

### 2.1. NSAIDs Discussed in This Review

The physico-chemical properties of NSAIDs are presented in Table 1. Diclofenac has been frequently detected in effluent wastewater samples due to its high resistance to biological treatment in sewage treatment plants [11,33].

Ibuprofen and its metabolites are predicted to be responsible for the endocrine-disrupting activity, and they have the same toxicology effect [35,36,37].

2-(3-benzoylphenyl) propanoic acid is ketoprofen, which is metabolized to release glucuronic acid. Naproxen is also a derivative of carboxylic acid, 2-(6-methoxynaphthalen-2-yl) propanoic acid, commonly used for the treatment of veterinary. Its acute toxicity is less than chronic toxicity [38].

Ibuprofen, isobutylphenyl propionic acid, is commonly detected in water samples due its high environmental importance [39].

### 2.2. Occurrence of NSAIDs in Surface Water, Influent, and Effluent Wastewater Samples

Wastewater treatment plants are a combination of physical, chemical, and biological treatment methods [40]. It uses the following four processes to treat wastewater: primary treatment, secondary treatment, tertiary treatment, and/or advanced treatment. The preliminary treatment stage by filtration and bar screen is the initial treatment step for removing coarse particles, sand, stone, and other big items that were present in wastewater. Additionally, it contributes to the exclusion of colloids and organic suspended particles. Microorganisms degrade organic molecules in the following “secondary step,” which is also known as the purification stage. Following these three processes, the tertiary treatment step employs an efficient treatment unit symbolized by advanced wastewater treatment and is employed for substances such as medicines that were not removed by the secondary step. The most popular methods for safely discharging treated effluent wastewater into water bodies include ozonation and UV irradiation [32]. While NSAIDs are polar chemicals that cannot be removed by wastewater treatment facilities, pharmaceuticals are polar to mid-polar organic molecules. Both adsorption onto suspended particles and hydrolysis are likely viable methods for removing these chemicals [41].

Table 2 enlists the presence of NSAIDs in surface water, groundwater, and influent and effluent wastewaters from WWTPs around the world. Of course, the concentration of NSAIDs in effluent WWTPs has changed and is not the same from country to country due to some considerations: (i) populations; (ii) type of treatment in WWTPs; (iii) the property of influent wastewater sample; (iv) season (rain/or dry); (v) unit water consumption; and (vi) unit amount wastewater per person. The order of concentration level of NSAIDs is arranged as follows: effluent from the pharmaceutical industry > effluent from hospital > effluent from WWTPs, according to the study reported previously [41]. The season has an impact on the detection of NSAIDs. It was reported that NSAIDs have been detected in high concentrations in cold weather compared to warm weather. However, this may be attributed to the fact that temperature and sunlight have a major role in degrading NSAIDs in water bodies [42]. In addition to the detection of the origin form of diclofenac, ibuprofen, and naproxen drugs in the effluent of WWTPs, their metabolites were also detected in water bodies. A few authors reported that three metabolites of diclofenac were detected in WWTPs effluent, which are 4′-hydroxydiclofenac, 5-hydroxydiclofenac, and acyl glucuronides [43,44,45].

1-hydroxyibuprofen, 2-hydroxyibuprofen, and carboxyibuprofen are metabolites belonging to ibuprofen that were present in WWTPs influent and river [62,63], whereas O-Desmethylnaproxen, a metabolite for naproxen, was also detected in surface water and effluents from Germany and Pakistan [64]. It was observed that some NSAIDs were present in influent WWTPs with concentrations lower than that of effluent WWTPs, probably related to the transfer of some conjugates into the origin compounds during the treatment process. Methodological errors and samples of influent and effluent were taken simultaneously without taking into account the hydraulic retention time [42,51].

In general, it was found that surface water samples and effluent wastewater samples contained lower levels of diclofenac and other chosen medications than influent wastewater samples. For instance, in an Australian research [58], influent and effluent wastewater samples had maximum naproxen concentrations of 67.6 and 0.161 µg/L, respectively. Similar to Portugal, there were 13 µg g/L of ibuprofen at its highest concentration in influent wastewater, as opposed to 0.025 µg g/L in effluent wastewater samples [50]. Therefore, while controlling pollution, these environmental changes in NSAID residues in the aquatic environment should be taken into account. Intense control and monitoring procedures are needed because more severe NSAIDs pollution is commonly reported in many countries. This higher NSAID abundance in treatment plants is likely caused by a number of factors, including the normally low average daily temperature, reduced UV intensity, and less precipitation [65,66,67]. However, multiple studies [68,69] discovered that the rainy season saw an increase in the environmental load of NSAIDs because of a very high flow rate brought on by rainfall that was more than the capacity of WWTPs.

**Figure 2:**
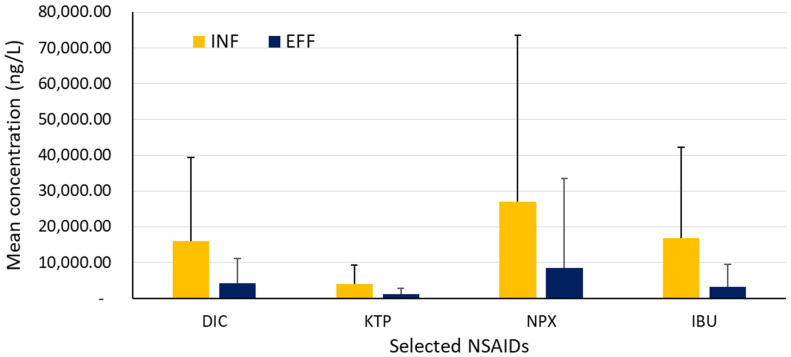
Mean concentration of diclofenac, ketoprofen, naproxen, and ibuprofen in influent and effluent wastewater samples in different countries. All resulted are cited from different sources: [27,32,42,46,47,48,49,50,51,52,53,54,55,56,57,58,59,60,61].

Other NSAIDs were also detected in groundwater, with concentrations ranging between 0.184 and 380 ng/L [4,42]. Ibuprofen was detected in groundwater at different levels according to the type of treatment and country. The range of concentration was 0.169–980 ng/L and 5.7–224 ng/L in Spain and Poland, respectively [4,42]. However, it was summarized that all results from different published studies show the mean concentration of the selected NSAIDs from different countries, as presented in Figure 2. Naproxen was detected at the highest concentration reaching up to 27 µg/L in influent wastewater; then, its concentration was 8.5 µg/L after treatment, then discharged to surface water. In general, the mean concentration of NSAIDs ranged between 4 and 27 µg/L in influent wastewater, but its concentration was reduced 3–4 times to 1.2–8.5 µg/L in the effluent wastewater. That means these compounds are still introduced into the surface water at high concentrations.

### 2.3. Evaluation of Wastewater Treatment Plants on Removal Efficacy of NSAIDs

Table 3 reported different removal efficiencies for NSAIDs from different wastewater plants worldwide. It could be expected that different removal efficiencies observed as NSAIDs have some different physico-chemical properties. Kummerova et al. (2016) reported that diclofenac had been eliminated from WWTPs in the range of 20–40% [70], while Aissaoui et al. (2017) investigated that the removal was more than 80% [30]. Elimination of ketoprofen from wastewater plants ranged from 38% to 100% [71,72]. Compared to diclofenac and naproxen, ibuprofen is highly eliminated in 90% [73].

However, EE-2 removal% reported that naproxen had been eliminated between 28% and 55% using (CAS + UV/chlorine) while it was completely degraded using (MBR + UV). This may be attributed to the effect of hydraulic retention time, which was between 1 and 3 days. In the same study, diclofenac was observed that it had achieved removal% ranging between 18% and 45% using conventional activated sludge combined with ultraviolet/chlorine.

In some cases, the concentration of diclofenac in effluent wastewater was higher than in influent wastewater [42,51,81]. The reasons are due to (i) improperly addressing the fluid dynamics of a WWTP; (ii) retransforming the conjugated compounds into the original compound due to biological processes in WWTPs; (iii) secondary treatment process releases the compounds-activated sludge; (iv) pharmaceutical compounds that may be released from fecal particles as the feces are being broken down by microbes. In South Africa, diclofenac, naproxen, and ibuprofen were eliminated from two wastewater plants by 80.5%, 95%, and 94.5%, respectively, on average, using a chlorination system. However, the removal% could be varied from one WWTP to another; diclofenac has achieved 69% elimination from Umbilo WWTP, while its removal was 92% from Kingsburgh WWTP using the same treatment system, “chlorination” [75].

In China, three different wastewater treatment plants were studied as one set, not individually. However, it was reported that diclofenac was eliminated by only 18.4% when the conventional treatments were applied, such as sedimentation, anoxic oxidation, and biologically aerated filters, while ibuprofen has achieved more than 80% removal under the same conditions [79]. Peng et al. (2019) reported that ibuprofen was eliminated by the biodegradation process. It was highly removed in the aerobic reactor compared to those systems that do not use oxygen but depend only on nitrogen source “anoxic system” [85]. The opposite was observed for diclofenac. It was not degraded in the aerobic system but was significantly eliminated in anoxic conditions [86,87].

A trickling filter is one of the treatments applied in WWTPs, which consists of a bed of rock, coke, polyurethane foam, or plastic media to form biofilm after transferring the sewage or wastewater downward the bed. This type exhibited acceptable removal of NSAIDs, followed by UV radiation. However, it showed high efficiency in enhancement after flowing the ozonated wastewater effluent because the elevated oxygen plays an important role in the sustainability of growth microbes [88]. Diclofenac, naproxen, and ketoprofen were eliminated effectively from effluent ponds as the germs degraded organic content after settling at a high retention time of more than 150 days [89]. Suitable results were obtained with the effluent ponds for the removal of NSAIDs, but they were not the preferred treatment process, as elevated hydraulic retention times were needed for this purpose.

### 2.4. Electrochemical Advanced Oxidation Processes

The occurrence of these pharmaceuticals in water sample bodies with no response from the traditional treatment plants requires looking for alternative treatment processes. Electrochemical oxidation processes (EOPs) are one of the main promising technologies for wastewater treatment and will be extensively published and applied in the near future. Hydroxyl radical (OH) is the strongest oxidizing agent that could be formed by anodic electrochemical oxidation processes (AEOPs). AEOPs are used frequently for the treatment of real wastewater samples and also for the elimination of different therapeutic classes of pharmaceuticals [1]. Oxidation based on OH has the advantage that it is non-selective, which means it produces a treated sample free of unwanted pollutants and makes it a promising technology for the treatment of biorefractory compounds in water [90,91]. According to the literature, many studies are published dealing with the removal of organic compounds from its aqueous solution, while some other reported studies focus on the treatment of the same organic compound in real wastewater, so it is worth evaluating the matrix effect during treatment of organic compound using an electrochemical process.

In the case of treatment of the pollutants from its aqueous solution, it could be clear to evaluate the mechanism of degradation reaction and kinetics followed by the possibility of the formation of unwanted products such as toxic or refractory transformed products. These products are negative indicators that tell us that it is difficult to apply this technology in that case. In the case of treatment of organic compounds in wastewater samples, the presence of other organic compounds and inorganic chloride ions may affect the efficiency of electrochemical treatment as the OH is consumed by these organic compounds. The characterization of the full wastewater sample is difficult as it has more than thousands of species to be analyzed and investigated.

### 2.5. Application of Anodic Electrochemical Oxidation Processes

Anodic electrochemical oxidation processes (AEOPs) are the oxidation that occurs on the anode electrode, achieving removal of the pollutants through direct oxidation “on the anode itself” or indirectly mediated oxidation “in the bulk solution” profile from its aqueous solution and/or wastewater. However, there are two types of strategy that could occur together or separately [92].

First, direct anodic oxidation: it is a very complex process, so it can be explained as (i) moving the organic pollutants from the bulk into the surface of the anode electrode; (ii) residing the organic pollutants on the surface of the anode, which is called “adsorption process”; (iii) direct electrochemical oxidation when the electron is transferred from the anode to the pollutants; (iv) desorption of the transformed products from the anode to the bulk. Second, indirect anodic oxidation process: it is totally different from the direct anodic oxidation process. In this case, some active oxidants are generated on the surface of the anode, then transferred into the bulk to attain the oxidation of organic pollutants. These oxidants are produced from the water or the added electrolyte.

Hydroxyl radical is one of these oxidants that could be formed from water, as follows:

(1) $$H_{2}O\;\to {\mathrm{OH}}_{\mathrm{ads}}^{\cdot }+H^{+}+e^{-}$$

(2) $${\mathrm{OH}}^{-}\;\to {\mathrm{OH}}_{\mathrm{ads}}^{\cdot }+e^{-}$$

Hydroxyl radical is the trademark of anodic oxidation, and it has the object of different studies [93,94]. Because of its high activity, hydroxyl radical has a very short lifetime, so the anodic oxidation near the surface could be limited, then called a heterogenous oxidation process. That is why distinguishing between direct and mediated oxidation anodic processes is difficult [95]. The promotion of other oxidants in the solution is possible due to the high oxidation capacity of hydroxyl radicals, then it participates in converting the direct oxidation process into a more efficient volumetric-oxidation process. Consequently, the formation of other oxidants, such as persulfates, peroxophosphates, and active chlorine (Cl_2_/H_2_O), is observed and reflects suitable efficiency in terms of remediation of pollutants.

In the electrochemical oxidation process, the type of electrode material is important. Some of them lead to complete remediation with the formation of few intermediates, while other types of electrode materials exhibit soft oxidation to the organic pollutants. It is well known that these types of electrodes are called “active vs. non-active, high-oxygen vs. low-oxygen overvoltage electrodes” based on their behaviors. High oxidation of pollutants comes from the presence of hydroxyl radicals in the solution during the electrochemical oxidation process. However, this suitable removal is observed through the promotion of the hydroxyl radical-mediated oxidation process and also generates highly stable oxidants.

Low-efficiency electrodes such as graphite and other carbon-based electrodes could degrade the organic pollutants softly with the production of great amounts of transformed products and also hardly degradable carboxylic acids [96]. Carbon-based materials electrodes can be electrochemically transformed into CO_2_ during the degradation of organic pollutants at high applied voltage. Equation 3 represents the formation of OH from water oxidation as it is not a free species attached to the surface of anode material (MO).

(3) $$M+H_{2}O\;\to \mathrm{MO}+2H^{+}+2e^{-}$$

Other carbon-source electrodes work as high-efficiency electrodes for the oxidation of organic pollutants, such as boron-doped diamonds (BDDs). This type of electrode participates in complete mineralization with the formation of a few intermediates during the electrochemical oxidation process. The BDD electrode is the only conductive electrode that forms OH at a high-efficiency anode heterogeneously from water oxidation, as presented in Equation (4), indicating that OH species are free close to the surface of the anode.

(4) $$M+H_{2}O\;\to M({\mathrm{HO}}^{\cdot })+H^{+}+e^{-}$$

It is very well known that biological and chemical oxidation treatment is dominant to be applied in wastewater treatment plants, while electrochemical oxidation is still in the first stages to meet the willing for application in plants. Many studies have investigated the treatment of anti-inflammatory drugs in synthetic wastewater to monitor the intermediates that are produced during the electrochemical oxidation of organic pollutants, which are sometimes more hazardous than parent compounds. In the present review, the authors focused on the electrochemical oxidation process using carbon-based anode as it is cheap and available.

Many published papers focused on carbon-based electrodes for the electrochemical treatment of selected NSAIDs from wastewater samples, as presented in Table 4. Brillas et al. (2005) is the first researcher to investigate using of BDD as an anode on paracetamol (acetaminophen) [97]. The compound was completely mineralized within pH ranging between 2.0 and 12.0 while using Pt as the anode; the mineralization was very poor. Brillas et al. (2010) also reported the degradation of diclofenac using a Pt/BDD anode. It was observed in complete degradation for diclofenac with the formation of some intermediates of carboxylic acids, such as oxamic and oxalic acids, which are the most persistent carboxylic acids. In comparison to the Pt anode, there was poor remediation with the formation of a great amount of malic, succinic, tartaric, and oxalic acids [98]. However, the mineralization rate was enhanced steadily with increasing current, although the efficiency decreased with increasing current due to the side reaction on the surface, such as oxidation of $\mathrm{BDD}({\mathrm{HO}}^{\cdot })$ to O_2_ (Equation (5)), production of hydrogen peroxide (Equation (6)), and destruction of hydrogen peroxide by a hydroxyl radical (Equation (7)).

(5) $$2\mathrm{BDD}({\mathrm{HO}}^{\cdot })\;\to 2\mathrm{BDD}+O_{2}\left(g\right)+2H^{+}+2e^{-}$$

(6) $$2\mathrm{BDD}({\mathrm{HO}}^{\cdot })\;\to 2\mathrm{BDD}+H_{2}O_{2}$$

(7) $$H_{2}O_{2}+\mathrm{BDD}({\mathrm{HO}}^{\cdot })\;\to \mathrm{BDD}(H_{2}O^{\cdot })+H_{2}O$$

“Peroxosulfates (S_2_O_8_)” and “peroxophosphates (P_2_O_8_)” are the oxidants that are produced during the electrochemical oxidation process by direct electron transfer or by the action of hydroxyl radicals, as explained in this set of Equations (8)–(13) [97]:

(8) $${\mathrm{SO}}_{4}^{2-}+{\mathrm{HO}}^{\cdot }\;\to \;{\left({\mathrm{SO}}_{4}^{-}\right)}^{\cdot }+{\mathrm{OH}}^{-}$$

(9) $${\left({\mathrm{SO}}_{4}^{-}\right)}^{\cdot }+{\left({\mathrm{SO}}_{4}^{-}\right)}^{\cdot }\;\to {\;S}_{2}O_{8}^{2-}+{\mathrm{OH}}^{-}$$

(10) $${\left({\mathrm{SO}}_{4}^{-}\right)}^{\cdot }+{\mathrm{HO}}^{\cdot }\;\to {\;\mathrm{HSO}}_{5}^{-}\;$$

(11) $${\mathrm{PO}}_{4}^{2-}+{\mathrm{HO}}^{\cdot }\;\to \;{\left({\mathrm{PO}}_{4}^{2-}\right)}^{\cdot }+{\mathrm{OH}}^{-}\;$$

(12) $${\left({\mathrm{PO}}_{4}^{2-}\right)}^{\cdot }+{\left({\mathrm{PO}}_{4}^{2-}\right)}^{\cdot }\;\to \;(P_{2}O_{8}^{4-})\;$$

(13) $${\left({\mathrm{PO}}_{4}^{2-}\right)}^{\cdot }+{\;\mathrm{HO}}^{\cdot }\to \;({\mathrm{HPO}}_{5}^{2-})\;$$

Diclofenac was degraded electrochemically using BDD as an anode in the presence of NaCl, then the formation of chlorinated byproducts is present. However, it was investigated that degradation of diclofenac at 30 mg/L increased with increasing applied voltage. After four hours, it had been mineralized with 72% at 4 volts. After degradation, new chlorinated byproducts appeared, such as 2,6-dichlorobenzenamine, 2,5-dihydroxybenzyl alcohol, benzoic acid, and 1-(2,6-dichlorocyclohexa-2,4-dienyl) indolin-2-one [99].

Ketoprofen was oxidized at 2 volts using BDD as an anode, and it was observed that the degradation increased with increasing current density. The mineralization current efficiency was influenced directly by the current density. It was observed that better mineralization (>80%) was accompanied by 4.4 mA/cm^2^ [100]. However, a sulfate ion as a supporting electrolyte ion was preferred in terms of TOC removal.

Zhao et al. (2009) investigated the electrochemical degradation of diclofenac in the presence of chloride ion using BDD as an anode [99]. It was observed that chlorides oxidized into hypochlorite ions (ClO) worked as an oxidation mediator or probably converted into perchlorate as presented in Equations (14)–(18), which is not enough to degrade the organic pollutants [101].

(14) $${\mathrm{Cl}}^{-}+{\;H}_{2}O\;\to \mathrm{HClO}+H^{+}+2e^{-}$$

(15) $${\mathrm{Cl}}^{-}+{\;\mathrm{HO}}^{\cdot }\;\to {\mathrm{ClO}}^{-}+H^{+}+e^{-}$$

(16) $${\mathrm{ClO}}^{-}+{\;\mathrm{HO}}^{\cdot }\;\to {\mathrm{ClO}}_{2}^{-}+H^{+}+e^{-}$$

(17) $${\mathrm{ClO}}_{2}^{-}+{\;\mathrm{HO}}^{\cdot }\;\to {\mathrm{ClO}}_{3}^{-}+H^{+}+e^{-}$$

(18) $${\mathrm{ClO}}_{3}^{-}+{\;\mathrm{HO}}^{\cdot }\;\to {\mathrm{ClO}}_{4}^{-}+H^{+}+e^{-}$$

Rather than BDD, graphite felt and graphite-PVC composite were investigated for electrochemical degradation of diclofenac. It was observed that the removal of diclofenac was 88% and 100% after 2 h and 45 min, respectively [102,103].

It was approved that the anodic oxidation kinetics of ketoprofen using BDD was fast and followed pseudo-first-order kinetics leading to complete mineralization for the parent compound and its intermediates. From the obtained results, the most significant parameters were pH and current intensity. The influence of pH was very significant, in which three different pHs were tested (3.0, 7.5, and 10.0). Faster TOC removal was observed with an acidic medium (pH 3.0), followed by pH 7.5, then pH 10.0 [104].

Domínguez et al. (2010) reported that ketoprofen has completely eliminated using BDD as an anode under optimum conditions: pH 3.99, oxygen flow rate 1.42 mL/min, current density 235 mA/cm^2^, and Na_2_SO_4_ 0.5 mol/L. Under the same conditions, the mineralization was just 36% in terms of COD, and the most significant factor was “current”, while the lowest was pH [105].

Another study reported by Feng et al. (2014) found that removal% of ketoprofen ranged between 45% and 100% based on the wide range of current density from 100 mA to 2000 mA, while high TOC% reaching up to 90% was achieved after 10 h using thin-film BDD provided with surface area 24 cm^2^ [104]. It seemed that thin-film BDD is more efficient than BDD as ketoprofen was completely mineralized “100% TOC” after 12 h [100].

In the case of ibuprofen, it was observed that the electrochemical degradation rate increased linearly with the current intensity but not proportionally as the current increased, which means progressive enhancement at a high current of the parasitic reactions; mainly oxygen evolution was achieved using BDD an anode, as shown in Equation (19) [106]:

(19) $$2H_{2}O\;\to O_{2}+4H^{+}+4e^{-}$$

In the same study, the presence of NaCl rather than Na_2_SO_4_ exhibited high removal percentage of ibuprofen due to the generation of a very active oxidizing agent (ClO^−^) from chlorine through these equations (20–22):

(20) $$2{\mathrm{Cl}}^{-}\;\to {\mathrm{Cl}}_{2}+2e^{-}$$

(21) $${\mathrm{Cl}}_{2}+H_{2}O\;\to \mathrm{HOCl}+H^{+}+{\mathrm{Cl}}^{-}$$

(22) $$\mathrm{HOCl}\;\to H^{+}+{\mathrm{OCl}}^{-}$$

In the presence of Na_2_SO_4_, it could also be explained that organic pollutants oxidized near the surface of the anode via hydroxyl radicals, so the degradation process depends on the limiting current intensity. Once the current exceeds the limiting line, the organic pollutants move from the bulk to the electrode surface, then oxidation occurs. Opposite to NaCl, the oxidation of organic compounds occurs in the bulk due to the direct reaction with active chlorine (HOCl/OCl^−^), so there are no mass transferring limitations to the anode surface [106].

In one study, the effectiveness of a novel three-dimensional graphene-coated nickel foam (Gr-NF) in the electro-peroxone method for the elimination of ibuprofen is investigated. The relative performance of the Gr-NF electrode was contrasted with that of a reticulated vitreous carbon electrode, which is frequently utilized. Within 8 h, the Gr-NF-based electro-peroxone system demonstrated a mineralization percentage of up to 75.0% TOC mineralization [107]. In order to completely mineralize ibuprofen within two hours, it was claimed that a carbon-polytetrafluorethylene cathode was employed to electrochemically produce H_2_O_2_ from O_2_ in the spared ozone generator effluent [108]. Using a BDD anode and a 3D carbon felt cathode, electrochemical advanced oxidation techniques were used to remove naproxen from the water via hydroxyl radicals ${\mathrm{OH}}^{\cdot }$. A pseudo-first-order reaction rate kinetic proved a suitable fit for naproxen’s deterioration. According to the reactive intermediates that were discovered, some of them are more hazardous than naproxen itself. However, the TOC removal procedure subsequently eliminated the generated hazardous intermediates [109]. Through the use of boron-doped diamond anodes and carbon felt cathodes in anodic oxidation procedures, the electrochemical degradation of ketoprofen has been investigated. Complete mineralization was attained through quick degradation of the drug’s parent molecule and its degradation intermediates. The obtained results demonstrated that increasing the applied current increased the rate of ketoprofen’s oxidative breakdown and mineralization of its aqueous solution. High-performance liquid chromatography analyses were used to pinpoint a number of chemical intermediates. Ion exclusion chromatography was used to track the synthesis, identification, and evolution of short-chain aliphatic carboxylic acids such as formic, acetic, oxalic, glycolic, and glyoxylic acids [104].

It is very well known that electrochemical degradation of NSAIDs, especially diclofenac, produces several byproducts, which are ended in CO_2_ and H_2_O. However, some products are dominant to be formed in different reported studies, such as “C_14_H_11_O_3_NCl_2_, C_14_H_11_O_3_NCl_2_, and C_13_H_9_ONCl_2_”, which are then converted into carboxylic acid [110,111].

The disadvantage of electrochemical degradation of NSAIDs is the generation of multi-chloro byproducts using graphite-PVC. Mussa et al. (2017) reported that during electrochemical degradation of diclofenac, penta-chloro-diclofenac products were formed, which is very interesting to be distinguished in LC-TOF/MS as chlorine has two different isotopes [103]. In the case of BDD as an anode, most of the byproducts were non-chlorinated byproducts, as observed with the electrochemical degradation of diclofenac [99,110], ketoprofen [104], ibuprofen [106,112], and naproxen [113] using sodium chloride as a supporting electrolyte. According to the literature, the disadvantages of using electrochemical oxidation processes include (i) high operational costs for electricity as required for all electrochemical methods, (ii) the likelihood that chlorinated organic compounds will form during the chlorine-mediated oxidation process, particularly for the treatment of wastewater with a high chloride content, are major drawbacks of the system, (iii) the need to conduct research on anode materials to determine the best electrode, (iv) the need for routine replacement of the “sacrificial electrodes” due to their oxidation-induced dissolution into wastewater streams, as well as the increase in conductivity of the remaining effluent, and (v) the sludge that is produced during the process will contain significant amounts of recalcitrant pollutant species that must be treated before disposal [114,115,116].

**Table 4:** Electrochemical degradation of selected NSAIDs using a carbon-based electrode.

Compound	Anode Material	Removal%	Main Byproducts	Observations	References
Diclofenac	BDD	100% (TOC) 100% (DIC)	- Carboxylic acids: malic acid; tartaric acid; succinic acid; oxalic acid; oxamic acid - Aromatic compounds - 2,6-dicloroaniline (C_6_H_5_NC_l2_) - 2,6-dichlorohydroquinone (C_6_H_4_O_2_Cl2) - 2-hydroxyphenyl acetic acid (C_8_H_8_O_3_) - 2,5-dihydroxyphenyl acetic acid (C_8_H_8_O_4_)	- The removal of TOC was achieved after 360 min under these conditions: 175 mg/L DIC, 0.05 M Na_2_SO_4_, pH 5.8 and current of 450 mA - The removal of diclofenac was achieved after 190 min under same above conditions - Thin-film BDD was 3 cm^2^ - Diclofenac concentration was monitored using HPLC - Carboxylic acid were analyzed using exclusion chromatography - Aromatic compounds were analyzed using GC-MS	[98]
	Graphite felt	88%	NS	- Graphite was as a cathode - This removal was the best compared to other cathode materials such as stainless steel and aluminum after 2 h - Electrochemical cell size was 500 cm^3^ - Surface area was 50 cm^2^ - Distance between electrodes was 1 cm - Electrochemical degradation was as three-dimensional electrochemical (3DE) reactor	[102]
	BDD disk	100% (DIC) 100% (TOC)	NS	- BDD electrode dimensions were: length 16 cm, height 4 cm and surface area 64 cm^2^ - Diclofenac was monitored using UV-visible spectrophotometer - Electrochemical cell size was 10 mL - Complete removal was observed for diclofenac and TOC after 50 and 250 min, respectively, under these conditions: 0.5 M NaClO_4_, 150 mg/L DIC, 330 mg L^−1^ COD	[117]
	Graphite-PVC	100% (DIC)	Several byproducts were generated in both positive and negative ionization modes, so we presented the main byproducts only: C_6_H_5_OC_l2_; C_11_H_8_O_4_NC_l3_; C_14_H_11_O_3_NC_l2_; C_13_H_8_ONC_l5_	- Electrochemical cell size was 100 mL - Graphite-PVC pellet was 10 mm in diameter - Diclofenac was monitored using LC-TOF/MS - Complete removal was observed after 40 min under these conditions: 6 volts, 4 g L^−1^ NaCl, 5 mg L^−1^ DIC	[103]
	BDD thin-film	100% (DIC)	Several byproducts were generated, so we presented the main byproducts only: C_14_H_11_O_3_NC_l2_; C_13_H_9_O_3_NC_l2_; C_13_H_9_O_2_NC_l2_	- Complete removal was achieved after 60 min at 292 mA cm^−2^ - Degradation efficiency was very influenced by current density - Thickness of BBD layer was 5–7 µm while its surface area was 24 × 50 mm^2^ - Diclofenac was monitored using HPLC	[110]
	Activated carbon fiber	100%	Several byproducts were generated, so we presented the main byproducts only: C_14_H_11_O_3_NC_l2_; C_13_H_11_O_2_NC_l2_ C_13_H_11_ONC_l2_, C_13_H_9_ONC_l2_	- The removal% observed was after a combination of permanganate oxidation and electrolysis using activated carbon fiber as a cathode - Complete removal was achieved after 10 min under these conditions: 20 µm DIC, current density 57 A m^−2,^ and permanganate dosage 100 µM - Experimental electrolysis reactor dimensions were 9 cm inner diameter and 12.5 cm height - Surface area of ACF was 17.5 cm^2^ - The distance between the two electrodes was 1 cm	[111]
	ND-BDD	72% (DIC)	Several byproducts were generated, so we presented the main byproducts only: C_14_H_9_O_3_NC_l2_; C_15_H_13_O_2_NC_l2_ C_14_H_10_O_2_NC_l3_	- Dimensions of electrode NB/BDD were 6 µm in thickness, 40 cm^2^, and (10 cm × 5 cm × 2 mm) cylindrical shape - Different electrolytes were used (Na_2_SO_4_, NaH_2_PO_4_, NaNO_3_) - Removal% was observed under these conditions: 50 µM DIC, current density 50 mA cm^−2^, 30 mM Na_2_SO_4_, and 30 min electrolysis time	[118]
	BDD	72% (TOC)	Several chlorinated and non-chlorinated byproducts were formed, such as: 2,6 -dichlorobenzeneamine; 1-(2,6-Dichlorocyclohexa-2,4-dienyl)indolin-2-one; 2,5-Dihydroxyl-benzeneacetic acid; 2.5-Dihydroxybenzyl alcohol; Oxalic acid; benzoic acid	- The reactor size is 100 mL - Surface area of the BDD anode is 12 cm^2^ - The concentration of diclofenac was 30 mg L^−1^ - GC-MS and LC-TOF/MS instruments were used to analyze the byproducts - TOC removal was achieved after 4 h of the electrochemical degradation process	[112]
Ketoprofen	BDD	100% (KTP) 36% (COD)	NS	- Electrode surface was 12.5 cm^2^ - Gap separation between two electrodes was 0.1 cm - Ketoprofen removal was monitored using high-performance liquid chromatography (HPLC) - Optimum conditions were pH 3.99, oxygen flow rate = 1.42 mL min^−1^, current density = 235 mA cm^−2^ and Na_2_SO_4_ = 0.5 mol L^−1^	[105]
	Thin-film BDD	45–100% (KTP) >90% (TOC)	Aromatic compounds: 3-acetylbenzophenone (C_15_H_12_O_2_) 3-hydroxyethyl benzophenone (C_15_H_14_O_2_) Benzophenone (C_13_H_10_O) 3-ethyl benzophenone (C_15_H_14_O) Carboxylic acid: Ten compounds, most of them are formic acid, acetic acid, malic acid, oxalic acid, and so on.	- Experimental conditions were 0.198 mM DIC, 50 mM Na_2_SO_4_ and 250 mL solution - Removal% was based on the wide range of current density from 100 mA to 2000 mA - High removal% in terms of TOC was achieved after 10 h - Ketoprofen was monitored using HPLC - Surface area was 24 cm^2^	[104]
	Thin-film BDD	100% (TOC)	NS	- Surface area was 11.25 cm^2^ - Electrochemical cell size was 250 mL - The distance between electrodes was 10 mm - Ketoprofen was monitored using a UV-visible spectrophotometer - Complete mineralization was observed after 12 h under these conditions: 0.1 M Na_2_SO_4_, 8.8 mA cm^−2^ current density, and 5 µM ketoprofen - Na_2_SO_4_ exhibited better removal compared to NaCl and NaNO_3_	[100]
Ibuprofen	Black carbon	60%	NS	- It was achieved after 60 min under these conditions: 5 mg L^−1^ IBU, 0.05 M Na_2_SO_4_, 0.03 A, and pH not changed - Removal was influenced by initial concentration, current, and Na_2_SO_4_ concentration - Ibuprofen was monitored by HPLC	[119]
	BDD	70–90% (TOC)	-different organic compounds were produced (data not shown). -carboxylic compounds: Maleic acid; oxamic acid; acetic acid; formic acid	- Surface area was 5 cm^2^ - 100 cm^3^ is the size of the electrochemical cell - Distance between two electrodes was 1 cm - 30 pharmaceuticals were spiked in wastewater samples and then treated - The observed removal% was provided after 300 min, and it was very influenced by the type of electrolyte and its concentration and current density	[120]
	BDD	60–95% (COD) 48–92% (TOC)	One compound has been reported: [2-(4-carboxycarbonyl)phenyl] propanoic acid (C_11_H_10_O_5_)	- Removal% was achieved after 6 h - Concentration of ibuprofen was 1.75 mM - Concentration of electrolyte (Na_2_SO_4_) was 0.035 M - 200 mL of ibuprofen solution was used	[112]
	BDD CNT GC	BDD = 50% CNT = 75 GC = 45	NS	- Three different carbon-based electrodes were provided - CNT exhibited better degradation in the presence of chloride and sulfate as support electrolytes - Removal% in terms of COD and TOC were 69 and 27.5%, respectively, using the CNT anode - Electrochemical cell was 100 cm^3^	[113]
	Thin-film BDD	>95% 91–96% (TOC)	Aromatic compounds: P-benzoquinone 4-isobutyhlphenol, 4-isobuthylacetophenone Carboxylic acid: Oxalic acid, glyoxylic acid, formic acid, acetic acid, and pyruvic acid	- High removal was observed after 90 min - Influence of pH, current, and initial concentration of IBU was investigated - Electrochemical cell with a 6 cm diameter and 0.2 L capacity was used - Presence of NaCl exhibited better removal% compared to Na_2_SO_4_ - High removal% of TOC was achieved after 8 h	[106]
Naproxen	MWCNTs-GCE	>80% (NPX) >60% (TOC)	Several byproducts were generated, so we presented the main byproducts only: C_13_H_12_O_2_; C_13_H_14_O_2_; C_12_H_10_O_2_; C_12_H_10_O_3_	- Electrode was prepared after deposition of multiwall carbon nanotubes (MWCNTs) on glassy carbon electrode (GCE) - Naproxen was monitored using HPLC - Removal% was observed after 25 min and 19 mg/L NPX	[121]

NS: not studied.

## 3. Possible Future Research Work

Future work on the electrochemical oxidation process could be explained as follows: The efficiency loss brought on by the impermeable oxide film generated on the cathode may be lessened by switching the polarity of the electrodes. Additionally, maintaining a constantly applied intensity rather than a constantly applied potential reduces the passivation of the electrodes even though it requires greater over-potentials to maintain the intensity while a passivating layer is formed. A further feature of this increase in over-potential is the potential for the direct oxidation of the organic matter in the effluent [122]. One way to get around some of the limitations, particularly the solid–liquid separation and the sludge elimination, is to integrate the EC process with other treatment techniques. To improve the recovery of fine particles and metal ions from wastewater, the EC technique can be built to incorporate membrane separation, reverse osmosis, electrofiltration, sludge dewatering, thermo-oxidation, and other standard technologies [123,124,125,126].

It is necessary to highlight the research gap in the present review:

- Application the electrochemical oxidation process in waste treatment plants rather than conventional oxidation process.
- Application electrochemical oxidation process on other therapeutic classes such as beta-blockers, lipid modifying agents, diabetics, and so on which is ready for human consumption.
- More environmental monitoring studies are required to give clear picture on the occurrence and the fate of NSAIDs in influent and effluent hospital, and leachate.
- Study the ecotoxicological risks for algae, crustaceans and fish based on detected concentrations of NSAIDs contaminants in surface water and other wastewater samples.

## 4. Conclusions

Although NSAIDs have been present in water for decades, their concentrations in the environment have only recently begun to be quantified and acknowledged as potentially hazardous to ecosystems. The water threat issue produced by NSAIDs in surface and ground waters has been acknowledged by many countries as an environmental problem.

Nevertheless, there is currently no legally regulated maximum of permitted concentrations of NSAIDs in the environment, despite their unknown impact on the aquatic environment and human health. The amounts of various NSAIDs detected in the influent and effluent of various WWTPs confirmed that many of these drugs are not effectively removed by the traditional treatments.

In some cases, the removal% was a negative value. This may be attributed to methodological errors, and samples of influent and effluent were taken simultaneously without taking into account the hydraulic retention time as well as the fact that the concentration in the effluent is higher than in the influent, as observed with diclofenac and ketoprofen in Algeria in which the removal% was −175% and −80%, respectively. Thus, conventional treatment systems are unable to completely eliminate a large amount of the NSAIDs present in wastewaters. More effective and specific treatments are required to reduce the environmental and potential impact of these pollutants. Among these treatments, the electrochemical degradation process is under research and has yet to be applied on an industrial scale since there is a lack of high-quality data on the mechanisms involved, the influence of operational variables, the reaction kinetics, and reactor design issues. However, carbon-based anodes are promising electrodes to be applied as they are available, cheap, and easy to obtain.

Recently, research on the degradation of NSAIDs using carbon-based anodes such as BDD, graphite, carbon black, and others has started. The literature claims that carbon-based anodes typically have a high ability to degrade NSAIDs, depending on the starting material, NSAIDs structure, and solution chemistry. Diclofenac, ketoprofen, naproxen, and ibuprofen can all be broken down electrochemically using a carbon-based anode according to research conducted in the last few years. The use of electrochemical processes to completely remove NSAIDs has recently attracted a great deal of research interest. Removal yields obtained from carbon-based anodes were high, which permits, in some cases, eliminating the pollutants completely but in terms of COD/TOC still not complete. However, achieving >90% TOC may require more than 10 h, as observed with ketoprofen using thin-film BDD as an anode. Most of the byproducts were chlorinated as NaCl was used as an electrolyte. The most common byproducts were aromatic byproducts, then mineralized into carboxylic acid before they were completely converted into CO_2_.

## Figures and Tables

**Table 1:** Physico-chemicals properties of selected NSAIDs.

Compound Name	Molecular Formula	pKa	LogK_ow_	Molar Mass (g mol^−1^)	Solubility (mg L^−1^)	Excretion%	References
Diclofenac	C_14_H_11_Cl_2_NO_2_	4.18	4.02	296.1	4.82	40%-bile 60%-urine	[4]
Ketoprofen	C_16_H_14_O_3_	3.88	3.12	254.28	120.4	NA	[34]
Naproxen	C_14_H_14_O_3_	4.19	3.10	230.26	144.9	95%-urine	[34]
Ibuprofen	C_13_H_18_O_2_	4.85	3.79	206.28	41.04	95%-urine	[34]

**Table 2:** Occurrence of selected NSAIDs in water bodies (surface water, effluent WWTP, influent WWTP, effluent hospital).

Name of Compound	Drinking Water/Tap Water/Ground Water (ng L^−1^)	Surface Water (ngL^−1^)	Influent WWTP (ng L^−1^)	Effluent WWTP (ng L^−1^)	Country	References
Diclofenac	0.184–380 ^a^	NS	NS	NS	Spain	[4]
	NS	NS	250	215	Spain	[32]
	NS	NS	NS	500–1020	Nigeria	[46]
	NS	NS	1240–53,000	<LOQ(1000)-15,000	South Africa	[47]
	NS NS	ND-10,000	22,300	19,000	South Africa	[48]
	NS	NS	44–230	22–33	Iran	[49]
	NS	NS	2120–62,722	1720–1997	Portugal	[50]
	NS	NS	990–2319	1616–2711	Algeria	[51]
	NS	NS	12,000	2000	Czech Republic	[52]
	NS	NS	152–185	100–131	Taiwan	[53]
	NS	NS	27–3160	23–570	Latvia	[54]
	114 ^b^	NS	556–4001	743–5402	Poland	[42]
Naproxen	<3–17 ^a^	NS	NS	NS	Nigeria	[4]
	NS	NS	99	108	Spain	[32]
	NS	23,800	159,000	91,100	South Africa	[48]
	NS	NS	<LOQ(770)-37,000	<LOQ(770)-4900	South Africa	[47]
	NS	NS	88–340	33–54	Iran	[49]
	NS	NS	11,044–13,093	25–41	Portugal	[50]
	NS	NS	1220–9585	ND-334	Algeria	[51]
	NS	35	32,900	4120	U.K.	[55]
	NS	43	NS	NS	China	[56]
	NS	<7	370	<13	U.K.	[57]
	NS	136	NS	1330	U.K.	[58]
	NS	80	430	110	Japan	[59]
	NS	NS	67,600	161	Australia	[60]
	NS	NS	350–4280	306–961	Latvia	[54]
**Ibuprofen**	0.16–988 ^a^	NS	NS	NS	Spain	[4]
	NS	NS	516	266	Spain	[32]
	NS	NS	NS	600–6600	Nigeria	[46]
	NS	445–689	1060	1380	South Africa	[48]
	NS	NS	<LOQ(3400)-72,000	<LOQ(3400)-21,000	South Africa	[47]
	NS	NS	233–1051	31–45	Iran	[49]
	NS	NS	11,044–13,093	25–41	Portugal	[50]
	NS	NS	1607–8612	341–431	Algeria	[51]
	NS	<9	ND	572	U.K.	[55]
	NS	325	NS	NS	China	[56]
	NS	75	390	75	China	[61]
	NS	NS	1643	210	Spain	[27]
	NS	<236	NS	460	U.K.	[58]
	NS	121	179	7	Australia	[60]
	NS	NS	64,000	15,000	Czech Republic	[52]
	NS	NS	108–28,500	152–1070	Latvia	[54]
	5.7–224 ^b^	NS	4198–10,864	24–644	Poland	[42]
Ketoprofen	NS	NS	451	318	Spain	[32]
	NS	Nd-437	3150	380	South Africa	[48]
	NS	NS	565	1035	Algeria	[51]
	NS	NS	39	200	U.K.	[55]
	NS	NS	510	177	Spain	[27]
	NS	NS	15,300	6	Australia	[60]
	NS	NS	6500	1000	Czech Republic	[52]
	NS	NS	377–9090	511–3730	Latvia	[54]
	13–167 ^b^	NS	73–322	1225–4030	Poland	[42]

NS: not studied. ^a^ groundwater; ^b^ tap water.

**Table 3:** Applied treatment of the selected NSAIDs from wastewater treatment plants.

Compound	Type of Treatment	Removal%	Country	Observations	References
Diclofenac	**CAS + UV/chlorine**	18–45	Spain	- This removal was achieved after combination with ultraviolet or chlorine - Hydraulic retention time was between 1 and 3 days - Samples were collected weekly within two months - All samples were stored at 4 °C without filtration	[74]
	**(HSF CW)**	42	Czech Republic	- Mean of four constructed wetlands located in the drinking water reservoir watershed - Samples were collected in six months - Every 4 h, samples were collected and then preserved in freeze	[52]
	NA	4–88	South Africa	- Range of removal refers to three selected WWTPs - Samples were collected within five months - Samples were filtered immediately, and pH was adjusted to 2.5 - After that, samples were stored at 4 °C	[75]
	**(GC) + (AT) + (SST) + (MP-SDU)**	80	South Africa	- Mean of two WWTPs receiving from hospitals - All four treatment processes were combined together - Samples were collected in duplicate for only two months, August and December 2015 - 10 mL of formaldehyde was added immediately to the sample and then stored at 4 °C	[76]
	Chlorination	81	South Africa	- Samples were collected weekly from two WWTPs within May 2016 - Samples were filtered first, then stored at 4 °C	[75]
	NA	30 and (−174)	Algeria	- Samples were collected from two different WWTPs; the second WWTPs exhibited high concentrations in effluent rather than influent - Samples were collected in November 2014 - Samples were filtered immediately; pH of the sample was adjusted to 2.5–3 and then stored at 4 °C	[51]
	Primary treatment: **(SGR + SD)** Secondary treatment: **(CAS + SD)** Disinfection system: **(UR)**	13–89	Turkey	- Samples were collected from one WWTP - Samples were collected every day in Summer (August) and Winter (January), so the removal% was written as a range - Samples were collected in amber glass bottles and then stored at 4 °C	[77]
	**(SR) + (CST) + (ASBT)**	39	Spain	- Samples were collected from one STP for five consecutive days from July 2003 to April 2004 - Samples were collected using amber glass bottled and then filtered before storage at 4 °C	[78]
	WWTP1: **(SD + BAF + UD)** WWTP2: **(SD + SOOD + UD)** WWTP3: **(SD + AA/O + SD + CD)**	18	China	- Removal% was calculated as an average of three WWTPs - Source of the sample is domestic and industrial wastewater - Samples were collected one time in four months in 2013	[79]
	**(CMBT)**	−41	Poland	- Samples were collected every 1–2 weeks from December 2012 to November 2013, covering four seasons - Removal% was calculated as average within one year of obtained results - Samples were collected from WWTP using amber glass bottles and then stored immediately at 4 °C	[42]
	**(AST)**	38	Italy	- Removal efficiency% was calculated as the average of four WWTPs in two months - Samples were collected every day for one week in February and May 2011 - Samples were collected from four WWTPs and it receive mainly domestic and industrial wastewater - Samples were filtered first and then adjusted to pH 3.6, and after that, stored at −20 °C	[80]
	**(PS + AST)**	−55	Spain	- Removal was calculated as the average of four WWTPs - Sixteen samples were collected from January 2008 to January 2009 - Samples were collected using amber glass bottles and then stored at 4 °C	[81]
	**(CAS)**	83	Portugal	- Removal was calculated as the mean of two WWTPs - Samples were collected monthly from October 2013 to June 2014 - Samples were collected in high-density polyethylene bottles and then filtered after that stored at −20 °C - Source of wastewater is domestic, hospital effluents, piggeries effluents, and landfill leachate	[82]
Ketoprofen	**(SR) +** Chlorination	88–90	South Africa	- Three different wastewater treatment plants - Samples were collected once per week within May 2016	[83]
	**(MBR)**	100		- Complete removal observed after combination with ultraviolet or chlorination - Hydraulic retention time was between 1 and 3 days - Samples were collected weekly within two months - All samples were stored at 4 °C without filtration	[74]
	**(HSF CW)**	52	Czech Republic	- Mean of three constructed wetlands located in drinking water reservoir watershed - Samples were collected in six months - Every 4 h, samples were collected and then preserved in freeze	[52]
	NA	−83	Algeria	- Samples were collected from two different WWTPs - In the first WWTPs, ketoprofen was not detected in influent and effluent. In the second WWTPs, ketoprofen exhibited a high concentration in effluent rather than influent - Samples were collected in November 2014 - Samples were filtered immediately; pH of the sample was adjusted to 2.5–3 and then stored at 4 °C	[51]
	- Primary treatment: **(S + GR + SD)** - Secondary treatment: **(CAS + SS)** - Disinfection system: **(UR)**	74–81	Turkey	- Samples were collected from one WWTP - Samples were collected every day in Summer (August) and Winter (January) so the removal% was written as range - Samples were collected in amber glass bottles then stored at 4 °C	[77]
	**(GC) + (AT) + (SST) + (MP-SDU)**	63	South Africa	- Mean of two WWTPs receiving from hospitals - All four treatment processes were combined together - Samples were collected in duplicate for only two months, August and December 2015 - 10 mL of formaldehyde was added immediately to the sample and then stored at 4 °C	[76]
	**(CMBT)**	94	Poland	- Samples were collected every 1–2 weeks from December 2012 to November 2013 covering four seasons - Removal% was calculated as average within one year - Samples were collected from WWTP using amber glass bottles and then stored immediately at 4 °C	[42]
	- WWTP1: **(SD + BAF + UD)** - WWTP2: **(SD + SOOD + UD)** - WWTP3: **(SD + AA/O + SD + CD)**	8	China	- Removal % was calculated as the average of three WWTPs - Source of the sample is domestic and industrial wastewater - Samples were collected one time in four months in 2013	[79]
	**(PS + AST)**	88	Spain	- Removal was calculated as the average of four WWTPs - Sixteen samples were collected from January 2008 to January 2009 - Samples were collected using amber glass bottles and then stored at 4 °C	[81]
	**(CAS)**	−59	Portugal	- Removal was calculated as the mean of two WWTPs - Samples were collected monthly from October 2013 to June 2014 - Samples were collected in high-density polyethylene bottles and then filtered after that stored at −20 °C - Source of wastewater is domestic, hospital effluents, piggeries effluents, and landfill leachate	[82]
Naproxen	**(CAS + UV/chlorine)**	28–55	Spain	- The removal was achieved after combination with UV or chlorine - Hydraulic retention time was between 1 and 3 days - Samples were collected weekly within two months - All samples were stored at 4 °C without filtration	[74]
	**(MBR)**	100		- Complete removal observed after combination with ultraviolet or chlorination. - Hydraulic retention time was between 1 and 3 days - Samples were collected weekly within two months - All samples were stored at 4 °C without filtration	[74]
	NA	16–87	South Africa	- The removal% was for three selected WWTPs within five months - Samples were collected within five months - Samples were filtered immediately, and pH was adjusted to 2.5 - After that, samples were stored at 4 °C	[75]
	Chlorination	95	South Africa	- Samples were collected weekly from two WWTPs within May 2016 - Samples were filtered first and then stored at 4 °C	[75]
	NA	73	Algeria	- Samples were collected from two different WWTPs in November 2014 - Samples were filtered immediately; pH of the sample was adjusted to 2.5–3 and then stored at 4 °C	[51]
	- Primary treatment: **(S + GR + SD)** - Secondary treatment: **(CAS + SS)** - Disinfection system: **(UR)**	99–88	Turkey	- Samples were collected from one WWTP - Samples were collected every day in summer (August) and winter (January), so the removal% was written as range - The source of wastewater is domestic, hospital, and industrial wastewaters - Samples were collected in amber glass bottles and then stored at 4 °C	[77]
	**(AST)**	47	Italy	- Removal efficiency% was calculated as the average of four WWTPs in two months - Samples were collected every day for one week in February and May 2011 - Samples were collected from four WWTPs, and they receive mainly domestic and industrial wastewater - Samples were filtered first and then adjusted to pH 3.6; after that, they were stored at −20 °C	[80]
	**(PS + AST)**	57	Spain	- Removal was calculated as the average of four WWTPs - Sixteen samples were collected from January 2008 to January 2009 - Samples were collected using amber glass bottles and then stored at 4 °C	[81]
	**(CAS)**	86	Portugal	- Removal was calculated as the mean of two WWTPs - Samples were collected monthly from October 2013 to June 2014 - Samples were collected in high-density polyethylene bottles and then filtered; after that, it was stored at −20 °C - Source of wastewater is domestic, hospital effluents, piggeries effluents, and landfill leachate	[82]
Ibuprofen	**(HSF CW)**	56	Czech Republic	- Mean of four constructed wetlands located in drinking water reservoir watershed - Samples were collected in six months - Every 4 h, samples were collected and then preserved in freeze	[52]
	NA	25–83	South Africa	- Range of removal refers to three selected WWTPs - Samples were collected within five months - Samples were filtered immediately, and pH was adjusted to 2.5 - After that samples were stored at 4 °C	[75]
	Chlorination	95	South Africa	- Samples were collected weekly from two WWTPs within May 2016 - Samples were filtered first and then stored at 4 °C	[75]
	NA	88	Algeria	- Samples were collected from two different WWTPs - Samples were collected in November 2014 - Samples were filtered immediately; pH of the sample was adjusted to 2.5–3 and then stored at 4 °C	[51]
	- Primary treatment: **(S + GR + SD)** - Secondary treatment: **(CAS + SD)** - Disinfection system: **(UR)**	97–80	Turkey	- Samples were collected from one WWTP - Samples were collected every day in summer (August) and winter (January) so the removal% was written as range - Samples were collected in amber glass bottles then stored at 4 °C	[77]
	**(SR) + (CST) + (ASBT)**	83	Spain	- Samples were collected from one STP for five consecutive days from July 2003 to April 2004 - Samples were collected using amber glass bottled and then filtered before storage at 4 °C	[78]
	**(CMBT)**	98	Poland	- Samples were collected every 1–2 weeks from December 2012 to November 2013, covering four seasons - Removal% was calculated as average within one year of obtained results - Samples were collected from WWTP using amber glass bottles and then stored immediately at 4 °C	[42]
	WWTP1: **(SD + BAF + UD)** WWTP2: **(SD + SOOD + UD)** WWTP3: **(SD + AA/O + SD + CD)**	83	China	- Removal% was calculated as the average of three WWTPs - Source of the sample is domestic and industrial wastewater - Samples were collected one time in four months in 2013	[79]
	**(PS + AST)**	87	Spain	- Removal was calculated as average of four WWTPs - Sixteen samples were collected from January 2008 to January 2009 - Samples were collected using amber glass bottles and then stored at 4 °C	[81]
	**(CAS)**	90	Portugal	- Removal was calculated as the mean of two WWTPs - Samples were collected monthly from October 2013 to June 2014 - Samples were collected in high-density polyethylene bottles and then filtered after that stored at −20 °C - Source of wastewater is domestic, hospital effluents, piggeries effluents, and landfill leachate	[82]
	**(AS) + (UD)**	17	Canada	- Removal efficiency was calculated as mean of three WWTPs - Samples were collected in July, October, and March to cover three seasons: summer, fall, and winter, respectively - Samples were collected every two weeks within one month - Collected samples were frozen before further analysis	[84]
	**(GC) + (AT) + (SST) + (MP-SDU)**	85	South Africa	- Mean of two WWTPs received from hospitals - All four treatment processes were combined together - Samples were collected in duplicate for only two months August and December 2015 - 10 mL of formaldehyde was added immediately to the sample and then stored at 4 °C	[76]
	**(AST)**	63	Italy	- Removal% was calculated as the average of four WWTPs in two months - Samples were collected every day for one week in February and May 2011 - Samples were collected from four WWTPs, and they received mainly domestic and industrial wastewater - Samples were filtered first and then adjusted to pH 3.6; after that, they were stored at −20 °C	[80]

Conventional activated sludge (CAS); horizontal subsurface flow (HSF); grit channels (GC); aeration tanks (AT); secondary sedimentation tank (SST); maturation pond sludge dewatering units (MP-SDU); primary treatment included screening, grit removal then sedimentation (SGR + SD); secondary treatment included conventional activated sludge and sedimentation (CAS + SD); disinfection system included ultraviolet radiation (UR); solid removal (SR); circular sedimentation tank (CST); activated sludge biological treatment (ASBT); WWTP1: sedimentation, biological aerated filter and UV disinfection (SD + BAF + UD); WWTP2: sedimentation, orbal oxidation ditches, sedimentation and UV disinfection (SD + SOOD + UD); WWTP3: primary sedimentation, anaerobic/anoxic/oxic, sedimentation and chemical disinfection (SD + AA/O + SD + CD); Conventional mechanical biological treatment (CMBT); activated sludge treatment (AST); primary settling, and activated sludge treatment (PS + AST); microbiological reactor (MBR); horizontal subsurface flow constructed wetland (HSF CW); primary treatment included screening, grit removal then sedimentation (S + GR + SD); secondary treatment included conventional activated sludge and sedimentation (CAS + SD); aerated sewage (AS).

## Data Availability

Not applicable.
